# Supplementary material for: Patterns of Intron Gain and Loss in Fungi
Source: PLoS Biol. 2004 Nov 30;2(12):e422. doi: 10.1371/journal.pbio.0020422 (PMC532390; doi:10.1371/journal.pbio.0020422)
Supplement: Table S1 — Also available at http://genes.mit.edu/NielsenEtAl/. (4.3 MB ZIP). [file pbio.0020422.st001.zip › NielsenEtAl/html/941.html]

AN0942.1.NCU00643.1.MG01231.1.FG00655.1


```
 CLUSTAL W (1.82) Multiple Sequence Alignments - Introns Inserted


Sequence 1: NCU00643.1	363 aa
Sequence 2: MG01231.1	372 aa
Sequence 3: FG00655.1	375 aa
Sequence 4: AN0942.1	386 aa
Alignment Length: 397 aa
Number Identitical Residues: 230 aa
Alignment Score (without introns) 10936


MG01231.1 	--MSATNGS---AAAAPSKKNIGVFTNPKHDLWINEAEPSLESVQKGSDELKEGQVTIAI
NCU00643.1	------------MASSASKTNIGVFTNPQHDLWISEASPSLESVQKG-EELKEGEVTVAV
FG00655.1 	MSPSAVEGNGVVDVKTTLKPNVGVYTNPNHDLWVAPAEPSAEAVKSG-SDLKQGEVSVAI
AN0942.1  	----------MATATVLEKPNIGVFTNPKHDLWVAESKPTLEEVKSG-ESLKPGEVTIEV
          	             .    * *:**:***:****:  :.*: * *:.* ..** *:*:: :

MG01231.1 	RSTGICG2SDVHFWHHGCIGPMIVREDHILGHESAGEIIAVHPSVTSLKVGDRVAVEPQV
NCU00643.1	RSTGICG2SDVHFWKHGCIGPMIVECDHVLGHESAGEVIAVHPSVKSIKVGDRVAIEPQV
FG00655.1 	RSTGICG2SDVHFWHAGCIGPMIVEGDHILGHESAGEVIAVHPSVSHLKVGDRVAIEPNI
AN0942.1  	RSTGICG2SDVHFWHAGCIGPMIVTGDHILGHESAGDVIAVAPDVTSLKVGDRVAIEPNV
          	******* ******: ********  **:*******::*** *.*. :*******:**::

MG01231.1 	ICYECEPCLTGRYNGCEKVDFLSTPPVPGLLRRYVNHPAVWCHKIGDMSWEDGAMLEPLS
NCU00643.1	ICNACEPCLTGRYNGCERVDFLSTPPVPGLLRRYVNHPAVWCHKIGNMSYENGAMLEPLS
FG00655.1 	PCGTCEPCLTGRYNGCETVQFLSTPPVPGMLRRYINHPAVWCHKIGNMSYENGALLEPLS
AN0942.1  	ICNACEPCLTGRYNGCEKVAFLSTPPVDGLLRRYVNHPAVWCHKIGDMSYEDGALLEPLS
          	 *  ************* * ******* *:****:***********:**:*:**:*****

MG01231.1 	VALAGIQRAGITLGDPVLVCGAGPIGLITLLCAKAAGACPLVITDIDDGRLKFAKELVPD
NCU00643.1	VALAGLQRAGVRLGDPVLICGAGPIGLITMLCAKAAGACPLVITDIDEGRLKFAKEICPE
FG00655.1 	VALAGMQRAEVRLGDPVLICGAGPIGLITLQCCAAAGASPIVITDISESRLAFAKELCPR
AN0942.1  	VSLAAVERSGLRLGDPCLITGAGPIGLITLLSARAAGATPLVITDIDEGRLKFAKELVPE
          	*:**.::*: : **** *: *********: .. **** *:*****.:.** ****: * 

MG01231.1 	VITFKVEGRPTAEDAAKSIVEAFG--------GVEPTLAIECTGVESSIASAIWAVKFGG
NCU00643.1	VVTHKVE-RLSAEESAKKIVESFG--------GIEPAVALECTGVESSIAAAIWAVKFGG
FG00655.1 	VITHKVE-RLSAEDSAKAIVKSFG--------GVEPTVAMECTGVESSIAAAVWSVKFGG
AN0942.1  	VRTYKVEIGFSAEETAEGIINAFNDGQGAGPDALRPRIALECTGVESSVASAIWSVKFGG
          	* *.***   :**::*: *:::*.....:....:.* :*:********:*:*:*:*****

MG01231.1 	KVFVIGVGRNEISLPFMRASVREVDLQFQYRYCNTWPRAIRLIQNKVIDLTKLVTHRFPL
NCU00643.1	KVFVIGVGKNEIQIPFMRASVREVDLQFQYRYCNTWPRAIRLVENGLVDLTRLVTHRFPL
FG00655.1 	KVFIIGVGKNEINIPFMRASVREVDIQLQYRYCNTWPRAIRLVENNVVDLSKLVTHKFKL
AN0942.1  	KVFVIGVGKNEMKIPFMRLSTQEIDLQYQYRYCNTWPRAIRLVKNGVINLQKLVTHRYAL
          	***:****:**:.:**** *.:*:*:* **************::* :::* :****:: *

MG01231.1 	EDALKAFETAADPKTGAIKVQIQSLE------------
NCU00643.1	EDALKAFETASDPKTGAIKVQIQSLE------------
FG00655.1 	EDAIKAFETSADPKTGAIKVMIQSLE------------
AN0942.1  	EDALKAFETAANPKTGAIKVQIMSSTADVEAASAGQKN
          	***:*****:::******** * *  :. .::::....
```
